# Liquid biopsy: a step closer to transform diagnosis, prognosis and future of cancer treatments

**DOI:** 10.1186/s12943-022-01543-7

**Published:** 2022-03-18

**Authors:** Saife N. Lone, Sabah Nisar, Tariq Masoodi, Mayank Singh, Arshi Rizwan, Sheema Hashem, Wael El-Rifai, Davide Bedognetti, Surinder K. Batra, Mohammad Haris, Ajaz A. Bhat, Muzafar A. Macha

**Affiliations:** 1 Department of Biotechnology, School of Life Sciences, Central University of Kashmir, Ganderbal, Jammu & Kashmir, India; 2 Laboratory of Molecular and Metabolic Imaging, Cancer Research Department, Sidra Medicine, PO BOX 26999, Doha, Qatar; 3 Department of Medical Oncology, Dr. B. R. Ambedkar Institute Rotary Cancer Hospital, All India Institute of Medical Sciences (AIIMS), New Delhi, India; 4 Department of Nephrology, All India Institute of Medical Sciences, New Delhi, India; 5 Department of Surgery, University of Miami Miller School of Medicine, Miami, FL USA; 6 Sylvester Comprehensive Cancer Center, Miller School of Medicine, University of Miami, Miami, FL USA; 7 Department of Veterans Affairs, Miami Healthcare System, Miami, FL USA; 8 Cancer Research Department, Research Branch, Sidra Medicince, Doha, Qatar; 9 Department of Internal Medicine and Medical Specialities, University of Genova, Genova, Italy; 10 College of Health and Life Sciences, Hamad Bin Khalifa University, Doha, Qatar; 11 Department of Biochemistry and Molecular Biology, University of Nebraska Medical Center, NE 68198 Omaha, USA; 12 Eppley Institute for Research in Cancer and Allied Diseases, University of Nebraska Medical Center , Omaha, NE 68198 USA; 13 Fred and Pamela Buffett Cancer Center, University of Nebraska Medical Center, University of Nebraska Medical Center, NE 68198 Omaha, USA; 14 Laboratory Animal Research Center, Qatar University, Doha, Qatar; 15 Center for Advanced Metabolic Imaging in Precision Medicine, Department of Radiology, Perelman School of Medicine at the University of Pennsylvania, Philadelphia, USA; 16 Watson-Crick Centre for Molecular Medicine, Islamic University of Science and Technology, (IUST), 192122 Awantipora, Jammu & Kashmir India

**Keywords:** Liquid biopsy, Cancer, Circulating tumor cells, Circulating tumor DNA, Tumor extracellular vesicles, Non-invasive tumor detection, Precision medicine Cancer diagnosis

## Abstract

Over the past decade, invasive techniques for diagnosing and monitoring cancers are slowly being replaced by non-invasive methods such as liquid biopsy. Liquid biopsies have drastically revolutionized the field of clinical oncology, offering ease in tumor sampling, continuous monitoring by repeated sampling, devising personalized therapeutic regimens, and screening for therapeutic resistance. Liquid biopsies consist of isolating tumor-derived entities like circulating tumor cells, circulating tumor DNA, tumor extracellular vesicles, etc., present in the body fluids of patients with cancer, followed by an analysis of genomic and proteomic data contained within them. Methods for isolation and analysis of liquid biopsies have rapidly evolved over the past few years as described in the review, thus providing greater details about tumor characteristics such as tumor progression, tumor staging, heterogeneity, gene mutations, and clonal evolution, etc. Liquid biopsies from cancer patients have opened up newer avenues in detection and continuous monitoring, treatment based on precision medicine, and screening of markers for therapeutic resistance. Though the technology of liquid biopsies is still evolving, its non-invasive nature promises to open new eras in clinical oncology. The purpose of this review is to provide an overview of the current methodologies involved in liquid biopsies and their application in isolating tumor markers for detection, prognosis, and monitoring cancer treatment outcomes.

## Introduction

**Fig. 1:**
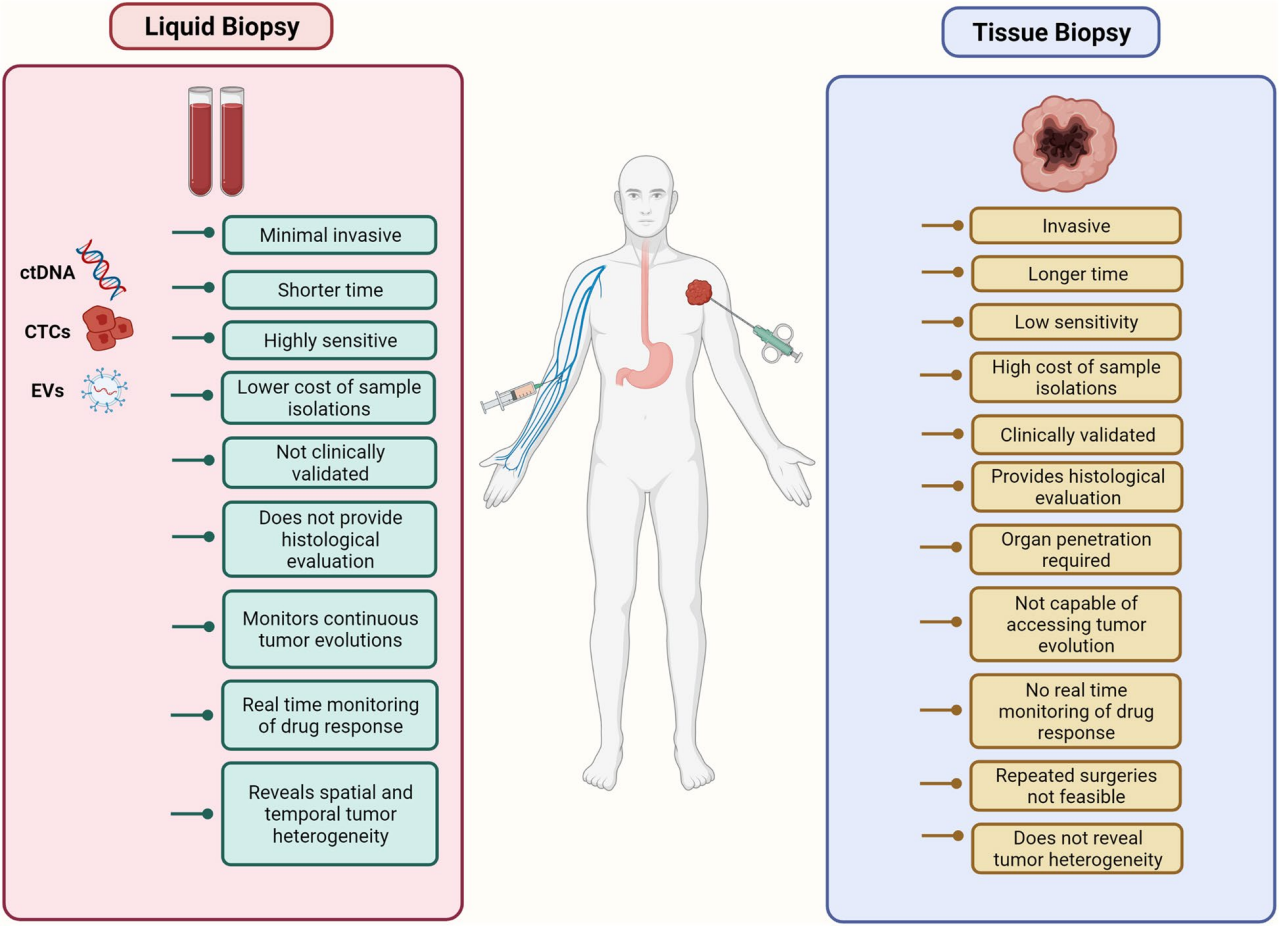
Comparison of traditional tissue biopsy and liquid biopsy. The schematic illustrates the advantages that liquid biopsies have gained over traditional invasive surgical methods over the past decade. Shown here are methods of extracting a test sample which usually includes a small tissue fragment in case of tissue biopsies and blood in LBs. Analytes that are isolated and monitored in LBs include ctDNA, CTCs, and tumor EVs

Molecular profiling of tumors obtained from individual patients has in recent years been shown to improve the selection of personalized cancer treatment therapies, patient responses, detection of drug resistance, and monitoring of tumor relapse [1, 2]. The standard method of profiling tumors initially involves obtaining resected tumor samples by invasive surgeries. The limitations to such invasive procedures include difficulty in acquiring tumor samples for both tumor quantity and quality (Fig. 1). Moreover, acquiring biopsy samples by invasive methods throughout treatment to monitor tumor response and relapse also poses a major challenge in tumor profiling [3]. Heterogeneity of resected tumor samples as a whole, also limits the use of invasive methods [4]. Additionally, in the case of metastasis, where tumors have spread and constantly evolve both spatially and temporally in response to treatment over time, multiple biopsies may be required as it is difficult to obtain a holistic image of a tumor [3]. Considering the challenges associated with traditional biopsies, recent oncology research has shifted its focus toward analyzing various biological fluids rather than whole tissues for tumor-derived components; a technique referred to as liquid biopsy (LB). Since blood contacts most of the tumors, LBs mostly involve blood sampling, although other body fluids like mucosa, pleural effusions, urine, and cerebrospinal fluid (CSF) are also analyzed [5]. Thus, LB provides enhanced sensitivity in diagnosis and ease of repeated sampling throughout treatment in a much more convenient and non-invasive way [6]. Moreover, studies have also focused on using LBs in the early detection of tumors [7].

**Fig. 2:**
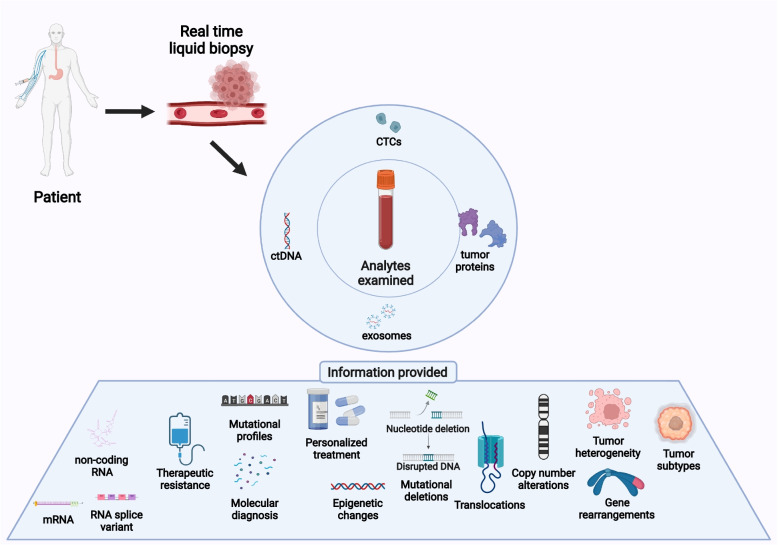
Entities analyzed in liquid biopsies and their application. The various analytes isolated from blood in LBs provide a wide variety of information regarding tumors. Each analyte has a specific application in tumor diagnosis, monitoring, and treatment as described

The technique is associated with both genomic as well as proteomic assessment of a wide array of tumor-derived moieties such as circulating tumor cells (CTCs), shed by both primary and metastatic tumors, circulating tumor DNA (ctDNA), tumor derived extracellular vesicles (EVs) that are membrane-bound subcellular moieties composed of nucleic acids/proteins; tumor educated platelets (TEPs), and circulating cell-free RNA (cfRNA), composed of small RNAs/miRNAs, etc. Taken together, these tumor-derived components can provide crucial longitudinal information and data for more accurate diagnosis by the pathologists regarding both primary and metastasized tumors. LBs encompass information like DNA mutations, copy number alterations (CNAs) of crucial genes [4], transcriptome/proteome profiling [8], epigenetic alterations [7], metabolite profiling [9], etc. (Fig. 2). Recent studies are also beginning to include bioinformatic tools in deciphering disease signatures using LBs [10].

This review provides an in-depth analysis of existing and newer methods of LBs involving isolation of CTCs, ctDNA, and EVs from the blood of patients with various types of cancers, along with citing applications of these extracted entities in detection, prognosis, and treatment of multiple types of cancers.

## Circulating tumor cells in liquid biopsies

Tumor cells were reported in the peripheral blood of patients as early as the 1860s, and significant improvements have been made ever since in obtaining CTCs from a heterogeneous population of blood cells [10]. CTCs are initially released from primary tumors in the tissue, travel through the circulatory system and account for the development of metastatic (or secondary) tumors at distant sites in the body [11]. In terms of numbers, their percentage in the blood is quite low, with nearly one CTC found per million leukocytes [12]. As far as morphology is concerned, studies have shown that CTCs vary in shape, depending on the stage and/or type of tumor [11]. Moreover, CTCs are known to develop into aggregates by attaching to cells like fibroblasts, platelets, etc., which have been reported to spread to more distant sites in the body relative to their isolated CTC counterparts. Such cellular aggregates are, thus, protected against oxidative stress and the surrounding immune system [13, 14].

CTCs have gained immense significance in detecting tumors, replacing invasive tissue biopsies not only due to their ease in sampling but also in providing data regarding tumor condition in a ‘real-time’ manner. CTC levels have been shown to change in a much more dynamic way, running parallel to the tumor condition with greater accuracy than usual biomarkers in the blood [15, 16]. CTC counts have also been reported to act as a better indicator of treatment response, with their reduced levels correlating with better overall survival (OS) in a large cohort of breast cancer (BC) patients [17]. CTCs, moreover, have shown promising results in the early diagnosis of several types of cancers like that of the lungs, albeit in a small group of patients with the chronic obstructive pulmonary disease [18]. Their diagnostic potential was confirmed by the presence of lung nodules and histotypic analysis of resected tissue samples later on. Interestingly, LBs using CTCs have more recently been reported to differentiate between the benign and malignant states of pulmonary lesions [19].

## Current technologies for isolation of CTCs from liquid biopsies

**Fig. 3:**
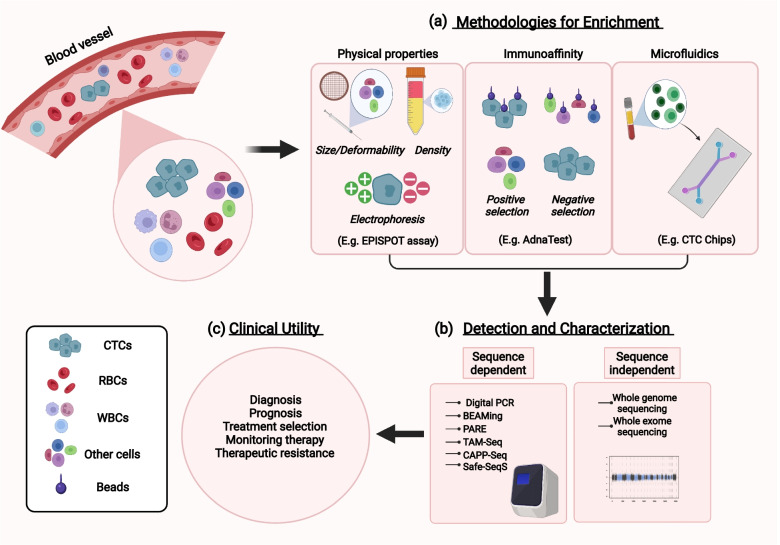
Overview of CTC isolation, detection, characterization and clinical utility. Schematic illustrating various methods of CTC isolation and detection. CTCs must be filtered out from the rest of the cells in the blood like WBCs, RBCs, etc. (**a**) Isolation and enrichment methods include assays based on physical properties (like size, density, etc.) of CTCs, their tendency to bind/not bind antibodies and microfluidic properties that assist in filtering out CTCs from rest of the cells in the sample like plasma or serum. (**b**) Detection and characterization of CTCs involve various techniques that employ primers requiring prior information of gene sequence (left) relative to those are exclusively deep sequencing-based (right). PARE: Personalized analysis of rearranged ends; TAm-Seq: tagged amplicon deep sequencing; CAPP-Seq: Cancer personalized profiling by deep sequencing; Safe-SeqS: safe sequencing system; BEAMing: beads, emulsion, amplification & magnetic and draw clinically relevant information regarding tumors. (**c**) The section summarizes the application of CTCs in clinical oncology

Various technologies have been used to selectively detect viable CTCs to obtain information regarding tumors (Fig. 3 & Table 1). One such example is the EPISPOT (EPithelialImmunoSPOT) assay that detects circulating tumor cells up to a single cell, and has been demonstrated by studies to be successful on a large number of patients with a wide variety of tumors such as those of BC, colon cancer (CL), prostate cancer (PCa), and melanomas [20]. The assay involves the use of membrane-bound antibodies against the epithelial cell adhesion molecule (EpCAM, or CD326) present on tumor cells and their subsequent culturing/expansion in both *in vivo* and *in vivo* conditions. The assay has prognostic relevance in characterizing the protein secretome of viable CTCs from breast cancer (BC) *in vivo* [21]. A similar positive selection cum enrichment technology for CTCs obtained from LB samples is the CellSearch system [15, 22]. The technology uses antibody-labeled magnetic beads to pull down CTCs with epithelial lineage markers (like EpCAM) (Table 1). The CellSearch system has been prominent in establishing a correlation between CTC cell counts and predicting patient survival in PCa [15]. There are, however, limitations to this assay, as not all CTCs in a heterogeneous population bear EpCAM markers and the fact that CTCs, once fixated, are not viable for further culturing and functional assays *in vivo* [23].

**Table 1:** Liquid Biopsy analytes and isolation technologies

LB analyte	Tumor type	Technology Used	Sensitivity/Detection limit	Basis of detection	Ref
CTCs	PCa	CellSearch system	73% for CTC ≥ 2 or 69% for CTC ≥ 5 per 7.5 ml	EpCAM	[15, 24]
	BC, PCa,CL	EPISPOT/S100-EPISPOT	48%; ≥ 2 CTCs	EpCAM, or CD326	[20, 25]
	PCa	AdnaTest	2 CTCs	EpCAM, PSA, and PSMA PCR	[26]
	PCa	AdnaTest	––	EpCAM and V7 variant PCR	[27]
	BC	CellSearch System	––	ER, BCL-2, EGFR 2, & Ki-67	[28]
		CTC-Chip	5–1, 281 CTCs per ml	tumor specific antigens	[29, 30]
		EasySep Depletion		CD45	[31]
		RosetteSep	2 CTCs/mL	CD45& DGC	[32, 33]
		SSA techniques	––	EpCAM	[34]
		Celsee systems	94%	Size differences and deformability	[35]
		ApoStreamTM	2 CTC/7.5 ml	Surface charge & polarizability	[36]
	Melanoma	DEPArrayTM System	––	Melan A^+^	[37]
ctDNA	BC, PCa, CRC	Droplet digital PCR	MAF detection < 0.1%	––	[38, 39]
		BEAMing	MAF detection ~ 0.02%	––	[3, 39–41]
	CRC, BC	PARE	ctDNA detection < 0.001%	––	[42, 43]
	OVC, BC	TAm-Seq/ eTAm-Seq	MAF detection ~ 2% MAF detection ~ 0.25%	–– ––	[39, 44]
	NSCLC	CAPP-Seq	MAF detection ~ 0.02%	––	[39, 45]
	BC	cMethDNA	––	––	[46]
	HEPC	MCTA-Seq	––	––	[47]
EVs		ExoMir™ kit	––	Nanomembrane ultrafiltration	
	OVA	SEC	––	Exclusion chromatography	[48]
	AML	Magneto-immunocapture	Higher purity, Lower yield	––	[49]
	PCa	Agglutination	––	Lectin	[50]
	PC	ExoChip	––	CD63 based immunochips	[51]
	Melanoma, PCa	––	––	CD63 and caveolin-1	[52, 53]
	PCa	––	––	prostate-specific transglutaminase	[54, 55]

*CTCs* Circulating tumor cells, *ctDNA* Circulating tumor DNA, *EVs* Extracellular vesicles, *BC* Breast cancer, *CL* Colon cancer, *CRC* Colorectal cancer, *OVC* Ovarian cancer, *PCa* Prostate cancer, *NSCLC* Non-small-cell lung cancer, *LADC* Lung adenocarcinoma, *AML* Acute myeloid leukemia, *HEPC* Hepatocellular carcinoma, *EPISPOT* Epithelial ImmunoSPOT, *MIC assay* Metastasis-Initiating-Cells, *DGC* Density gradient centrifugation, *PARE* Personalized analysis of rearranged ends, *MCTA-Seq* Methylated CpG tandem amplification and sequencing, *SEC* Size exclusion chromatography, *SSA* Selective size amplifications, *BCL-2* B-cell lymphoma 2, *EGFR-2* Epidermal Growth Factor Receptor 2, *ER* Estrogen receptor 1, *AR* Androgen receptor, *RB1* Retinoblastoma 1, *MED1* Mediator complex subunit 1, *GAS6* Growth arrest-specific 6, *MAF* Mutant allele fraction

Yet another immunomagnetic-based enrichment assay of CTCs from LBs is the AdnaTest. In addition to the EpCAM-labeled ferromagnetic beads used in the CellSearch system, AdnaTest includes a polymerase chain reaction (PCR) step to detect tumor-specific mRNA transcripts [26]. For example, in the case of CTCs from PCa, the assay consists of a PCR step using primers against prostate-specific markers like PSA, PSMA, etc. [26]. The test was also extended to detect tumor-specific splice variants of transcripts in CTCs enriched from LB samples. For example, the AdnaTest has been demonstrated to detect the *androgen receptor splice variant-7 (AR-V7)* transcript overexpressed in PCa, lacking the ligand domain constitutively expressed as a transcription factor [27]. The ligand-independent *AR-V7* variant, thus, results in a subsequent upregulation of AR modulated genes [56]. Furthermore, these recent studies have correlated detection of AR-V7^+^ variant CTCs with increased aggressiveness, poor prognosis and resistance to several chemotherapeutic drugs (enzalutamide and abiraterone) in cancers [27]. The clinical remarks from these studies, that non-AR directed therapies would better treat AR-V7 + individuals, were confirmed by subsequent studies of Onstenk et al. that demonstrated the proficient use of cabazitaxel in PCa [57]. Furthermore, CTCs isolated by the CellSearch System targeted against markers such as estrogen receptor, B-cell lymphoma 2 (*BCL-2*), Human Epidermal Growth Factor Receptor (*EGFR*) 2, and *Ki67* are crucial in the development of novel CTC-Endocrine Therapy Index, a parameter predicting response to endocrine therapy in patients with BC [28].

Apart from using antibody-labeled beads for the positive selection of CTCs, alternative approaches like microfluidic devices have also been used to select CTCs in various types of cancers [58, 59] (Fig. 3). Devices like the ‘CTC-Chip’, which contains thousands of small antibody-labeled microposts, have been used to capture CTCs bearing specific tumor antigens from the LB blood sample [29]. Newer designs of ‘CTC-Chips’ have been demonstrated to employ better patterns of microgrooves, which seem to increase the contact time between antibody-labeled microposts and CTCs, improving cellular entrapment. CTCs filtered off from LB samples by the chip are then imaged and analyzed [30, 60].

Moreover, functional assays like the Metastasis-Initiating-Cells (MIC) assay analyze the invasive properties of CTCs obtained from LB into the surrounding matrix *in vivo*, assisting in their further characterization [61]. These analyses, therefore, aid in providing a detailed picture of tumor staging/subtypes and in designing novel personalized therapeutic drugs against tumors [62]. In addition to general nuclear and surface-specific markers targeting CTCs, counterstain markers that target cells in exclusion to CTCs such as white blood cells (WBCs), platelets, red blood cells (RBCs), etc., can also be used to enrich CTCs from blood samples. The prominent markers selected for counterstains include CD45/CD66b (granulocytes), CD235a (RBCs), CD41/CD61 (platelets), CD4/CD8 (lymphocytes), CD11b/CD14 (macrophages) and CD34 (hematopoietic progenitors/endothelial cells) [63–66]. Technologies like the EasySep Depletion Kit (StemCell Technologies) use CD45-labeled magnetic beads to negatively select WBCs, depleting them from the LB samples [31]. Similar negative depletion technologies have been developed by others [67, 68]. Other examples, like the RosetteSep (StemCell Technologies) method, use an additional density gradient centrifugation step for further CTC enrichment [32]. The limitations of negative selection methods are that other blood components, like endothelial cells that are CD45^−ve^ can also crossover, and there is a greater risk of CTC loss in bulk WBC pulldowns [31].

Although expressed differentially on cancer cells, tumor-associated markers have a pitfall of being lost over time in CTCs due to cellular changes and dedifferentiation, even in aggressive forms of tumors [69]. Apart from differential expression of antigen markers, several other techniques have been reported that have enabled proficient identification and isolation of CTCs, such as differences in their physical properties vis-a-vis WBCs. Separation methods based on size exclusion have been reported to separate CTCs (mean diameter—15.6 μm) from WBCs (diameter range of 7–15 μm), as the former were relatively larger [70]. The limitation was that in many cases CTCs are nearly the same size as WBCs, resulting in a loss of up to half the CTCs in techniques relying solely on size exclusion [71]. In addition to this, smaller CTCs were also reported to be correlated with greater metastases in PCa [72]. These limitations have been overcome by using selective size amplifications techniques of CTCs that artificially increase their size using microbeads labeled with anti-EpCAM antibodies, improving cell recovery and purity [34]. Differences in deformability between CTCs and normal blood cells have also been exploited to isolate CTCs by allowing passage through microfluidic channels. These studies have revealed that the relative differences in deformability between WBCs and CTCs are much more prominent (CTCs being more deformable than WBCs) than between CTCs themselves, thus enabling proficient detection and isolation of CTCs [73]. Newer methods have also been developed more recently (Celsee systems) that use both size differences and deformability to isolate and analyze CTCs. These systems use microfluidic devices that house fluidic channels, along with capture chambers that entrap relatively larger tumor cells, whereas normal cells like WBCs pass through [35]. Furthermore, since the system entraps unlabelled cells, it can also be used for downstream analysis of CTCs by various techniques such as immunocytochemistry or in situ hybridization methods such as ‘FISH’. These microfluidic-based devices (Celsee systems) have indeed shown better sensitivity in detecting CTCs, as evident by their higher CTC counts relative to technologies based on cell surface markers (CellSearch system). The propensity to lose small CTCs still exists in these systems, though. Similar antibody independent technologies that are employed to isolate and recover CTCs from blood include the ApoStream^TM^device, which analyzes the differences in surface charge and polarizability between CTCs and normal blood cells [36]. Subsequent studies have again demonstrated these dielectrophoresis-based systems result in enhanced detection and recovery of CTCs in PCa relative to the surface marker-based systems (CellSearch system) [74].

Post-enrichment technologies, like the DEPArray™ System, have also been demonstrated to successfully isolate and recover single CTCs (like Melan A^+^ melanoma cells) from LB samples of whole blood [37]. Here next-generation sequencing (NGS) analysis is carried out directly on CTCs using technologies such as the Ion Torrent PGM™ system, composed of the Ion AmpliSeq™ Cancer Hotspot Panel, which provides enhanced mutational analysis and avoids the use of error-prone methods like whole genome amplification (WGA), thus improving screening accuracy [37].

## Application of liquid biopsy-derived CTCs in clinical oncology

Mutational profiling of CTCs for tumor-related genes provides crucial information regarding tumor characterization and predicting the outcome of therapeutic responses. For example, secondary point mutations in the *epidermal growth factor (EGFR) gene*, where a threonine residue replaces methionine (T790M), have been associated with tumor relapse and confer resistance to otherwise effective therapeutic agents such as gefitinib and erlotinib in patients with lung adenocarcinoma [75]. Interestingly, such mutations may positively predict therapeutic responses against newer irreversible EGFR tyrosine kinase inhibitors [76]. Similar mutational profiling of CTCs for EGFR-related genes like *KRAS* and *PIK3CA* indicates therapeutic outcomes in colorectal cancer (CRC) [77]. These studies reveal that heterogenic expression and genomic alterations in these genes across individuals may account for their varied response rates to treatment in CRC [77]. Thus, the molecular and genetic profiling of CTCs isolated from patients assists in detecting ever-evolving changes in the tumor genotype in a “real-time” manner (otherwise not detectable by conventional tissue biopsy) and in devising newer and more effective therapeutic responses.

CTCs isolated from LBs can provide valuable data on epigenetic changes of various tumor-relevant genes in cancers. Epigenetic alterations, like DNA methylation, in the promoter regions of tumor/metastasis suppressor genes, such as *SOX17, BRMS1*, and *CST6* in EpCAM^+^ CTCs isolated from individuals with BC, are known to be correlated with enhanced tumor metastasis and poor prognosis [78]. Similar alterations in the methylation profiles of genes like *VEGF and SFRP2*, associated with angiogenesis, have been observed in CTCs isolated from PCa and CRC patients, respectively [61, 79]. Moreover, studies have also revealed CTCs isolated from LBs to be an efficient diagnostic tool relative to tissue biopsies in detecting epigenetic changes in cancer-relevant genes [79]. In addition to prognosis and diagnosis, epigenetic alterations, such as estrogen receptor 1 methylation, have been demonstrated to be indicative of treatment resistance to chemotherapeutic regimens like everolimus and exemestane in BC patients [80].

Similarly, methylation profiles of non-coding RNAs (ncRNAs) associated with epithelial-to-mesenchymal transition, such as miR-200, are upregulated in CTCs isolated from PCa patients [81]. Changes in the epigenetic signatures of various genes in CTCs, thus, act as biomarkers assisting in prognosis, monitoring tumor response, and reflecting corresponding changes in cellular mechanisms suggestive of tumor metastasis. It is of interest to note that epigenetic alterations displayed by CTCs (or ctDNA, as discussed later in the review), though quite valuable in cancer prognosis, do not always reflect the state of primary tumors which constantly evolve [82].

CTCs can also be used *in vivo* for the generation of patient-derived tumor models that assist in treatment. It has been shown that BC xenografts composed of luminal BC CTCs contain MICs that are shown to induce metastasis of bone, liver, and lungs in mice [83]. The study revealed a correlation between CTC surface markers like EPCAM^+^, CD44^+^, CD47^+^and MET^+ve^, with an increase in the number of metastatic sites and reduced survival rates, thus providing data for the development of better diagnostic tools for the treatment of metastatic BC [83]. Similar studies on LC CTC-derived xenografts have provided better insights into therapeutic drug trials, disease prognosis, and resistance mechanisms [84]. To overcome the problem of low numbers of CTCs, studies have also focused on generating continuous cell lines from CTCs. For example, the CL cell line CTC-MCC-41 established from patients has been shown to have a stable phenotype sharing properties with its primary tumors, thus allowing functional studies and both *in vivo* and in vitro drug therapeutic trials to be carried out [85].

## Circulating tumor DNA in the liquid biopsies

Early studies by Leon et al. first demonstrated that patients with pancreatic cancer (PC) had elevated levels of ctDNA in their sera which seemed to decrease post-therapy [86]. Soon after, studies revealed that it was not only the levels that were altered in tumors but also their sequences, with ctDNA samples from plasma of patients with tumors reporting mutations in oncogenes such as *KRAS* [87]. Moreover, studies have established ctDNA (or chromosomal fragments) to be transferred horizontally via uptake of apoptotic bodies released by tumor cells, resulting in genetic changes in the host cell, promoting cellular transformation and metastasis [88]. It must be noted that ctDNA accounts for only 0.1–10% of the total circulating cell-free DNA (cfDNA), whose normal plasma levels range from 10–100 ng/ml [89]. Physiological states such as inflammation or exercise are also known to enhance cfDNA levels, which are not always reflective of underlying malignancy [90]. Moreover, ctDNA levels in the plasma vary subject to tumor load, tumor stage, and therapeutic response [91]. In addition to quantification, clinical application of ctDNA in precision medicine also allows analysis of ctDNA variants in the plasma. Recent studies have shown ctDNA to differ in length from the circulating cfDNA pool, with reports indicating ctDNA fractions in patients with cancer to be 20–50 base pair, relatively shorter than cfDNA [92].

## Current technologies for ctDNA analysis from liquid biopsies

The relative comparison of data from several sources is still a major hurdle in ctDNA analysis in clinical usage, as methods of sample handling, ctDNA isolation, and analysis have not yet been fully standardized, and a complete analytical consensus is lacking. The observed variations in both the quantity and the quality of ctDNA must be attributable to biological changes accompanying the tumor and not to the artifacts generated due to variation in sample handling. Studies have therefore focused on analyzing the effects of various ‘preanalytical’ factors like clotting [93], DNA leakage from WBCs and hematopoietic cells [93], freeze-thawing, DNAse activity of blood [94], PCR compatibility of reagents [94], the time-lapse between blood drawing and analysis [93], and temperature [95] in ctDNA analysis. Limitations associated with isolation and analysis of extremely low levels of ctDNA have to a large extent been reduced by ever-evolving technological applications [4, 96].

Two major types of approaches have been considered for ctDNA analysis: targeted approaches that focus on specific gene rearrangements or gene mutations in particular genomic regions that act as ‘hotspots’ for variation in a given tumor type, or untargeted approaches that offer a broader analysis and monitoring of the tumor genome, providing information on nucleotide alterations, copy number aberrations, chromosomal alterations, etc., independent of any prior data on molecular alterations (Fig. 3). Targeted approaches include PCR-based methods such as droplet digital PCR and BEAMing that have shown remarkable sensitivity of 1 to 0.001% in detecting somatic point mutations (Fig. 3) [97, 98]. Droplet digital PCR involves partitioning the sample DNA (target and background DNA) into numerous independent partitions or droplets. The target sequence is then amplified by end point PCR in each droplet and relative fractions of positive and negative droplets counted (fluorescent probes) that provide relative quantification of target samples [3, 98, 99]. Digital PCR has been shown to detect ctDNA in more than 75% of patients with advanced CRC, BC, and PC and to a good extent in patients with localized tumors [38, 100]. BEAMing (beads, emulsions, amplification, and magnetics), on the other hand, is a modification of emulsion PCR where several different templates are amplified within a single tube, each in different compartments (or emulsion droplets) but along with primer bound beads that are recovered with the help of a magnetic field or centrifugal force [3, 40]. PCR-based assays that detect genomic rearrangements explicitly associated with the tumor genome have shown promising results in sensitivity and specificity using ctDNA. Assays such as personalized analysis of rearranged ends (PARE), which uses primers flanking the breakpoint region, have been shown to successfully detect mutant ctDNA (rearranged sequences) at levels as low as 0.001% in plasma samples of patients with CRC and BC [42, 43]. PARE analysis of ctDNA thus assists in monitoring disease burden and the development of tumor-specific biomarkers in patients with solid tumors [43]. Numerous NGS-based methods have recently been developed that offer a relatively broader screening of the genomic regions, along with better resolutions in detecting mutations in ctDNA samples. Assays such as tagged-amplicon deep sequencing (TAm-Seq), developed by Forshew et al., can detect ctDNA mutations in plasma with very low allelic frequencies (~ 2%) and with high sensitivity (> 97%) [44]. Various sequence-specific primers first amplify multiple regions of the targeted area in the genome to allow the representation of various alleles in the template material, narrowing down the pool of amplified products. These diverse products are again amplified for enrichment, tagged with adaptors, and sequenced [44]. These studies have identified mutations in the tumor suppressor p53 and EGFR regions in plasma of patients with ovarian cancer (OVAC), otherwise not detectable by invasive solid biopsies [44]. Moreover, TAm-Seq has also assisted in the longitudinal screening of tumor mutations over several months in plasma of patients with BC. Similar deep sequencing methods like CAPP-Seq have been developed by Newman et al. that allowed the detection of ctDNA mutant fractions as low as 0.02% with high specificity (~ 95%) in patients with non-small-cell lung cancer (NSCLC) [45]. ctDNA quantified by CAPP-Seq analysis was shown to be better in correlating to tumor burden, detecting residual disease and accessing an early tumor response than traditional radiographic methods [45]. Tagged complementary oligonucleotide probes that can be recovered are used to target specific regions of DNA.

In contrast to the targeted approaches discussed above that focus on primer-specified regions, untargeted methods are relatively more comprehensive about analyzing the tumor genome. In this context, methods such as shotgun massively parallel sequencing of ctDNA from plasma have been shown to provide whole-genome profiling for copy number alterations (CNA) and mutations in patients with hepatocellular carcinoma (HEPC), BC, and OVAC [101]. Similar whole-genome profiling of plasma ctDNA using high-throughput IlluminasMiSeq has been shown to reveal various CNAs (androgen receptor amplification) and chromosomal rearrangements (*TMPRSS2-ERG* fusion; 8p loss/8q gain) in patients with castration-resistant and castration-sensitive PCa [46]. Whole-genome analysis using massively parallel sequencing of plasma ctDNA has also enabled the detection of similar alterations in patients with CRC and BC [102].

## Application of liquid biopsy-derived ctDNA in clinical oncology

Studies have revealed that ctDNA provides a much more holistic view of tumor characteristics and progression emanating from primary and metastasized tumor foci [103, 104] (Table 2). Moreover, mutations undetected by conventional tissue sampling have been screened using ctDNAs from LBs [105]. Genome sequencing of ctDNA has also assisted in detecting tumor-specific copy number alterations of genes in PCa and reveals a constantly changing nature of cancer cell genomes where gene amplifications play crucial roles in cancer progression [106]. ctDNA profiling has also enabled tracking of clonal variations in patients with CRC, assisting in real-time monitoring of tumor progression and therapeutic resistance against EGFR blockade [107]. Similarly, clonal profiling of tumor cells using ctDNA has also been studied in PCa where androgen receptor mutations have been screened that emerge against chemotherapeutic regimens like abiraterone or prednisolone [108]. ctDNA profiles of clones in these studies reveal both spatial and temporal tumor heterogeneity arising due to differences in resistance mechanisms at different tumor sites.

**Table 2:** Clinical applications of LB in various cancers

LB entity	Cancer	Analysis	Diagnosis provided	Ref
EVs	PCa		glypican-1 (GPC1), KRAS mutation	[109]
	PCa		miRNAs, CD44v6, Tspan8, EpCAM and CD104	[110]
	NSCLC	miR-23b-3p, miR-10b-5p and miR-21-5p	Noninvasive biomarker	[111]
		miR-125b-5p	Predicting improved T-cell activity	[112]
	NSCLC	miR-146a-5p	Predicting chemosensitivity	[113]
	melanoma	miR-211-5p	Predicting resistance to vemurafenib	[114]
	melanoma	PD-1 and CD28	Predicting resistance to ipilimumab	[115]
CTCs	LADC	EGFR mutation	Predicting gefitinib and erlotinib	[76]
	CRC	KRAS, PIK3CAmutation	Predicting therapeutic response	[77]
	BC	Promoter methylation of SOX17, BRMS1and CST6	Poor prognosis	
	PCa and CRC	Promoter methylation of VEGF and SFRP2	Predicts angiogenesis	[61, 79]
	BC	ER 1 methylation	Predicts everolimus and exemestane resistance	[59
	BC	EPCAM^+^, CD44^+^, CD47^+^& MET^+^ expression	Predicts metastasis	[83]
ctDNA	CRC	Genomic profiling	Tracking clonal variations and therapeutic response	[107]
	PCa	AR mutations	Predicting abiraterone or prednisolone response	[108]
	B cell lymphoma	DNA profiling	Determine tumor subtypes	[116]
	OVC, CRC	DNA profiling	Poor clinical outcome	[117–119]
	BC, CRC	DNA profiling	Residual disease and relapse	[120, 121]
	CRC	KRAS, NRAS, and BRAF mutations	Predicting panitumumab and cetuximab response	[122, 123]
	Solid tumors	mutations in PIK3CA, RB1, MED1, GAS6 and EGFR	Predict response to paclitaxel, cisplatin, tamoxifen, lapatinib and gefitinib	[124]

*CTCs* Circulating tumor cells, *ctDNA* circulating tumor DNA, *EVs* Extracellular vesicles, *BC* Breast cancer, *CL* Colon cancer, *CRC* Colorectal cancer, *OVC* Ovarian cancer, *PCa* Prostate cancers, *NSCLC* Non-small-cell lung cancer, *LADC* Lung adenocarcinoma, *AML* Acute myeloid leukaemia

Moreover, ctDNA genotyping is known to assist in determining tumor subtypes in patients with B cell lymphoma, thus assisting in predicting clinical outcomes and personalized treatment [116]. Relative to ctDNA, a higher number of CTCs or multiple solid tumor biopsies would be required to access similar outcomes, thus highlighting the proficiency of ctDNA biopsies. Moreover, unlike CTCs, ctDNAs are known to act as biomarkers indicative of tumor volume, as revealed by studies in OVAC and lung cancer (LC) [117, 118]. The prognostic significance of ctDNA in cancer progression and its therapeutic response has been revealed in several types of cancers such as OVAC [117], LC [118], and CRC [119], where its presence correlates with relatively poor clinical outcomes and tumor relapse. In addition to prognosis, monitoring ctDNA profiles in patients with BC and CRC has also enabled the detection of residual disease post-therapy and the risk of relapse, thus allowing therapy modification and avoiding overtreatment [120, 121]. Plasma-Seq analysis of ctDNAs reveals wide variety of mutations or aberrations that act as predictive resistance markers against therapies in various forms of cancer. For instance, *KRAS-, NRAS, and BRAF*-associated mutations in plasma ctDNA of metastatic CRC patients drive primary resistance five to six months post-anti-EGFR regimens like panitumumab and cetuximab [122, 123]. Similarly, mutations in PIK3CA, retinoblastoma 1, mediator complex subunit 1, growth arrest-specific 6 and EGFR confer resistance against drugs like paclitaxel, cisplatin, tamoxifen, lapatinib, and gefitinibin in advanced cancers [124]. Interestingly, even though resistance mechanisms that develop over time have different origins genetically, they seem to converge at crucial signaling foci. Thus, there are numerous applications of ctDNAs in diagnosis, prognosis, therapy and drug resistance associated with precision oncology.

Apart from mutations, epigenetic modifications such as methylation patterns of tumor-derived ctDNA have been described as biomarkers for various cancers [7, 125]. Similar to screening ctDNA mutations, methods for detecting ctDNA methylation are classified into two types: localized site-specific targeting of selected regions of the genome and a holistic genome-wide approach [96]. Various PCR-based methods require prior knowledge about the targeted region, such as methylation-specific PCR (MSP) [126], quantitative multiplexed MSP [127], and methylation on beads [128, 129], which are known to assist in detecting methylated DNA sequences. Fluorescence-based real-time modifications to conventional MSPs have also been shown to facilitate the quantitative detection of methylation patterns [130]. Assays such as cMethDNA, a modified version of QM-MSP developed by Fackler et al., are quite proficient in detecting late-stage BCs and monitoring tumor progression and treatment response, as well as disease recurrence [46]. The assay provides enhanced methylation signals of novel biomarkers in the sera of BC patients and better sensitivity and reproducibility than conventional QM-MSPs [46]. Methylation levels were shown to correlate with treatment response and act as a connecting link between ctDNA and its parent tissue of origin [46].

Interestingly, these studies found epigenetic signatures to be retained in the sera of patients several years post-diagnosis of disease. Genome-wide approaches such as Shotgun massively parallel bisulfite sequencing have been shown to detect ctDNA methylations with better sensitivity and specificity [7]. Similar genome-wide approaches such as methylated CpG tandem amplification and sequencing have been described to detect numerous hypermethylated CpG islands in ctDNA samples as low as 7.5 pg in plasma of patients with HEPC [47].

## Extracellular vesicles from tumor liquid biopsies

**Fig. 4:**
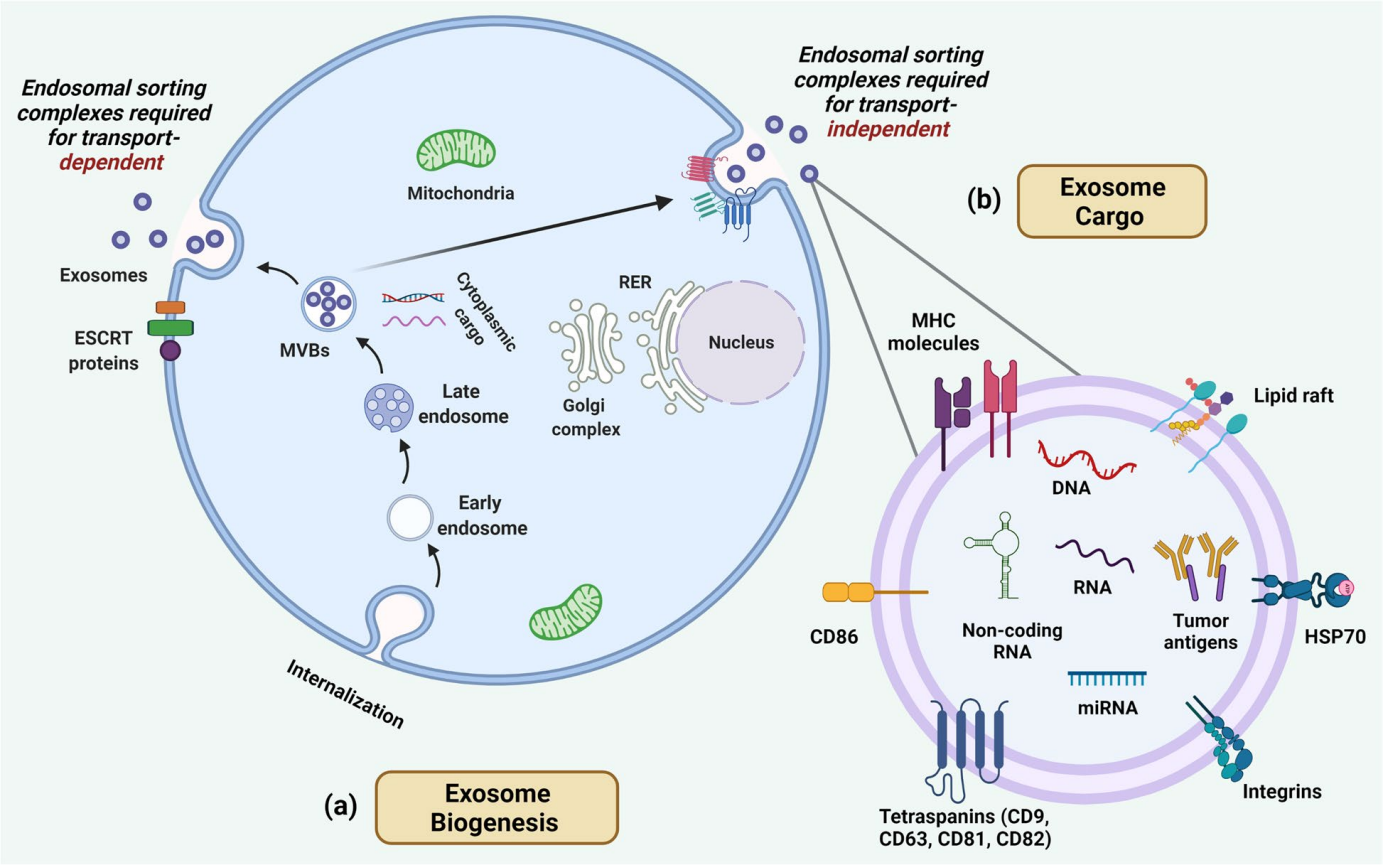
Extracellular vesicle biogenesis and cargo. (**a**) The schematic illustrates the synthesis of EVs (via endosomal sorting complex required for transport (ESCRT) dependent or independent pathway), inside the cell that begins by inward budding of the plasma membrane. Early endosomes formed to take up cytoplasmic cargo that includes biomolecules like DNA, RNA, and proteins that play a role in cell-to-cell communication. Multivesicular bodies, thus, formed containing a wide variety of cellular cargo, soon merge with plasma membrane releasing EVs. Cell-specific surface antigens are known to be tagged along in certain cases while EVs emerge from a cell. (**b**) The figure depicts the wide array of biomolecular cargo (both internalized and surface bearing) that EVs carry and exploited as markers in the characterization of tumors. Tumor derived EVs bearing numerous markers (as depicted) provide efficient noninvasive ways of LBs that offer real-time monitoring of tumor progression and treatment

Yet another candidate for LBs are extracellular vesicles (EVs), small 30–100 nm, membrane-bound, saucer-shaped vesicles secreted by cells and found in various body fluids such as plasma, urine, cerebrospinal fluid (CSF), saliva, etc. [131, 132]. Formerly regarded as cellular waste products assisting in removing undegraded endosomal or lysosomal components, EVs have now been established to play crucial roles in various forms of cell-to-cell communication [125]. EVs cargo comprised of diverse biomolecules like DNA, RNA, protein, etc., has been established to play crucial roles in intercellular communication [53, 133–135] (Fig. 4). Most importantly, tumor-derived EVs have gained immense attention as studies have described their roles in promoting tumor growth, epithelial to mesenchymal transition, metastasis, immunosuppression, angiogenesis, etc. [53, 132–134, 136, 137]. Since tumors have been shown to shed EVs profusely, their numbers in the plasma of patients with tumors reach extraordinary levels [134]. As tumor-derived EVs contain crucial cargo such as tumor-derived DNA, mRNA, ncRNAs, proteins, etc. [138] (Fig. 4), their analysis offers significant insights into tumor monitoring, prognosis, and therapeutic response.

## Current methodologies for the isolation of tumor extracellular vesicles from liquid biopsies

Several techniques have been described that effectively isolate EVs from varied cellular debris, exploiting various unique physical and biochemical properties that assist in EVs isolation. Preparative ultracentrifugation is known to be one of the most common EVs isolation methods, accounting for nearly half of all the EVs isolation methods currently employed [139, 140]. The technique exploits differences in particulate density, shape, and size. Variations that prevent EVs loss and contamination and improve EVs purity, like differential, isopycnic, and moving zone ultracentrifugation, have been described by various studies [50, 141, 142]. Other size-based EVs isolation methods include ultrafiltration, which employs membrane filters with predefined molecular weight cutoffs [143]. Although ultrafiltration-based isolation methods dispense with the use of specialized equipment like centrifuges, the vesicles are vulnerable to breakage or deformation due to force used, thus, affecting downstream analysis [144]. In this context, nanomembrane ultrafiltration concentrators have been shown to successfully isolate urinary EVs from as little as 500 µl of sample and assist in diagnosing renal complications [145]. Commercial kits like ExoMir™ (Bio Scientific) employ syringe filters to capture EVs from cell-free fluids like sera, CSF, and cell culture media. RNA extraction reagents are then used to lyse and release exosomal contents, further processed for qRT-PCR analysis. Variations such as sequential filtration that use electron microscopy and mass spectrometry for downstream analysis allow EVsisolation with better purity and integrity [146]. Similar size-based EVs isolation methods include size exclusion chromatography, where EVs are separated from the rest of the components in the sample by being excluded from the pores in the stationary phase and eluted earlier than the rest of the fractions [147]. For example, EVs secreted by mesenchymal stem cells in response to myocardial infarction have been isolated by such size-exclusion methods [148]. Similar size exclusion fractionation methods have been utilized for isolating tumor-derived EVs capable of T-cell suppression from ascites of OVAC patients, which are then confirmed for biomarkers by Western blots [48]. Moreover, studies have also shown that size exclusion chromatography, in combination with ultracentrifugation, improves urinary EVs yield and assists in detecting prognostic biomarkers capable of predicting nephrotic disease outcomes [149]. Newer size-based EVs separation methods include techniques such as flow field-flow fractionation, involved in isolating EVs from neural stem cells [150], and hydrostatic filtration dialysis, effective in separating and enriching urinary microvesicles to form relatively larger sample volumes [151]. In addition to size-based isolation methods, immunoaffinity-based assays such as ELISA have been reported to isolate and analyze EVs from body fluids such as plasma, urine, CSF, etc. These methods exploit the presence of membrane-bound surface biomarkers that are either specifically expressed or overexpressed on EVs [139]. Moreover, RNA extracted from EVs isolated by immunoaffinity-based assays displayed better yield than conventional size-based methods like ultracentrifugation, capable of extracting exosomal RNA from as small as 100 μl of sample [139]. Variations of these methods like the magneto-immunocapture technique that use antibody-coated magnetic particles have been demonstrated to display yields 10–15 times better than UC [139]. Tetraspanins, such as CD63, highly expressed on EVs membranes, are known to be exploited for selective enrichment of EVs from complex sample mixtures using these techniques (ThermoFisher). Moreover, CD34 + blast-derived EVs captured by similar magneto-immunocapture techniques have been predicted to act as biomarkers and to be useful in monitoring disease progression and therapeutic response in acute myeloid leukemia [49]. Interestingly, EVs isolated by these methods were shown to be biologically active and capable of mediating immune suppression. Various studies have also highlighted variants of magneto-immunocapture-based techniques that offer better capture efficiency and sensitivity in isolating tumor EVs [152–155]. Precipitation methods that use polymers, like polyethylene glycol, have been successful in isolating EVs from biological fluids [156]. Commercial isolation kits based on precipitation of EVs include ExoQuick PLUS (System Biosciences) that are capable of precipitating EVs from serum, plasma, etc. in a relatively shorter time frame and with reduced carryover contaminants. EVs so isolated are then analyzed for their protein content by Western blots and for RNA by qRT-PCR [157, 158]. EVs isolation kits (ThermoFisher, CUSABIO, iZON, qEVSingle, 101Bio) have also been developed that cater to a wide range of samples like urine, saliva, CSF, ascetic fluid, and amniotic fluid. Pre- and post-isolation steps for EVs purification are also included that get rid of non-EVs contaminants like lipoproteins and polymeric materials, respectively [139]. In addition to polymers, lectins with a high binding affinity to oligosaccharide residues on EVs membranes have also been reported to be quite successful in isolating urinary EVs [159]. miRNA profiles of EVs precipitated by such lectin-based agglutination methods have been suggested to act as crucial biomarkers in diagnosing PCa [50]. Furthermore, microfluidics-based enrichment methods such as those developed by Wang et al. use ciliated micropillars that exclusively entrap EVs with the exclusion of other extracellular vesicles, proteins, and debris [160]. Likewise, commercially available microfluidic devices such as ExoChip, developed by Kanwar et al., successfully isolate EVs from sera of patients with PCs for miRNA analysis [51]. These devices capture EVs using a CD63 antibody-coated immunochip, followed by membrane-specific fluorescent staining. Exosomal cargo is analyzed by either Western blot (for proteins) or RT-PCR (RNA). Similar immunochip-based assays employing antibody-labeled magnetic beads use plasma samples as low as 30 μl with an assay time < 100 min for EVs enrichment and analysis [161]. Despite the rapid advances made in EVs isolation and enrichment as described above, the technologies are still limited in various clinical applications. Issues such as pretreatment of samples, isolation efficiency, standardization, EVs heterogeneity and more importantly, yield of exosomal cargo pose some major challenges to researchers working in this area. Nevertheless, the significance of EVs in clinical applications such as tumor diagnosis, monitoring and treatment cannot be underestimated given their huge significance as a minimally invasive technique in these areas, as evidenced by various sources described below.

## Applications of tumor derived extracellular vesicles from liquid biopsies in clinical oncology

Post enrichment and purification, EVs analyzed for their protein or nucleic acid cargo are efficacious as diagnostic and prognostic markers in a wide variety of cancers. EVs expressing biomarkers such as CD63 and caveolin-1 are known to act as potential indicators of melanoma [52]; those enriched with migration inhibitory factor act as predictive markers of liver metastasis in patients with PC [53]; and those tumor-derived EVs with markers like prostate-specific transglutaminase and stem cell antigen are indicative of tumor burden in patients with PCa [54, 55]. Recent studies have highlighted the role of tumor-derived EVs in diagnosing early-stage PCs. Tumor-derived EVs in these studies were enriched with biomarkers like glypican-1, a cell surface proteoglycan, along with harboring *KRAS* mutations [109]. Apart from membrane-bound markers, exosomal cargo like DNA and RNA are also known to provide crucial information for the diagnosis and therapeutic response of patients in various types of cancers. Upregulated miRNA cargos of miR-1246, miR-4644, miR-3976, and miR-4306 were reported to act as highly sensitive and minimally invasive biomarkers in patients with PC [110, 162]. These studies indicated a similar diagnostic potential for exosomal proteins like CD44v6, Tspan8, EpCAM and CD104. EVs enriched with miRNAs such as miR-23b-3p, miR-10b-5p and miR-21-5p in plasma of patients have been reported to act as significant non-invasive biomarkers for NSCLC [111]. LB profiles of exosomal miRNAs also assist in determining the efficacy of treatment against cancers. In post-treatment plasma EVs, downregulation of immunosuppressor miRNA, like miR-125b-5p, has been correlated with improved T-cell activity and better response to immunotherapy [112]. A similar correlation between exosomal miRNAs and the efficacy of chemotherapeutic drugs has been described in the case of cisplatin used against NSCLC, where elevated levels of exosomal miR-146a-5p were shown to result in increased chemosensitivity of NSCLC to the chemotherapeutic drug [113].

Conversely, miR-146a-5p expression levels were shown to drop in both NSCLC cell lines and EVs in cisplatin resistance. Furthermore, upregulated miRNA signatures of miR-211-5p in melanoma and melanoma-derived vesicular secretome have been suggested as indicative of therapeutic resistance developed against BRAF inhibitors such as vemurafenib, used in treating metastatic melanomas [114]. Corroborating with this, these studies also demonstrated an inverse correlation between elevated miR-211-5p levels and reduced sensitivity to *BRAF* inhibitors *in vivo*. Exosomal cargo such as DNA also assists in providing firsthand information on tumors. Mutations in exosomal DNA at regions harboring tumor-relevant genes like *KRAS and p53* have been reported to be indicative of PCs [163]. In addition to tumor-derived EVs, studies have suggested that even serum EVs derived from immune cells act as biomarkers that assist in predicting a clinical response against chemotherapeutic drugs. Elevated levels of surface markers PD-1 and CD28 on T-/dendritic cell-derived EVs in melanoma patients have been correlated with improved clinical response to chemotherapeutic drugs like ipilimumab [115]. More recently, studies have described small extracellular vesicles (sEVs) derived from serum to have potential roles in monitoring tumors of the central nervous system [132].

Apart from miRNAs, other non-coding RNAs like lncRNAs and circRNAs present in tumor-derived EVs have also been shown to play promising roles in the diagnosis and monitoring of tumors. For instance, circRNAs like *circ_0044516* have been observed to be upregulated in PCa derived blood exosomes [164, 165]. Silencing the expression of *circ_0044516* in PCa cells corroborated with a reduction in growth and metastasis of cancer cells. Similar roles of non-coding RNAs such as exosomal lncRNAs have been described in PCa [166]. Exosomal lncRNA H19 is known to be upregulated in serum in cases of bladder cancer, thus, having a potential role as a prominent diagnostic marker [167]. Similarly, elevated levels of lncRNAs PCA3 and BCAR4 have been reported in the sera of patients with CRC [168]. Serum exosomal lncRNAs *ENSG00000258332.1*, *LINC00635* and *HEIH*, on the other hand, have potential diagnostic roles in LB for hepatocellular carcinoma [169, 170]. In addition to diagnosis, exosomal lncRNAs also find application in tumor prognosis, such as lncRNA *MALAT1* in epithelial ovarian cancer (EOVAC) and lncRNA *MALAT1*, PCAT-1 in BC [171, 172]. Furthermore, exosomal lncRNAs also find crucial application in monitoring drug resistance of tumors indicating their potential clinical use in cancer therapy. For example, tumor derived lncRNA *H19* displays positive correlation with gefitinib resistance in patients with non-NSCLC [173]; lncRNA *PART1* with similar geftinib resistance in esophageal squamous cell carcinoma (ESCC) [174]; lncRNA ARSR with sunitinib resistance in advanced renal cell carcinoma (RCC) [175]; lncRNA *UCA1* expression with cetuximab-resistant CRC cells [176]. Exosomal lncRNA mediated LB, therefore, offers tremendous application in real-time monitoring of tumor diagnosis, progression, and recurrence in a wide variety of tumors.

## Non-blood candidates in liquid biopsies

In addition to circulatory fluids like plasma or serum discussed above, various other body fluids like saliva, urine, etc. have also been shown by numerous studies to have significant application in liquid biopsy. Saliva, for instance, offers practical advantages with regard to ease of access, non-invasiveness, and cost effectiveness in sampling, even more so than plasma or serum [177]. Salivary molecular diagnostics have evolved rapidly over the past decade with great potential in cancer detection, monitoring, and development of point-of-care medicine [178]. Novel electrochemical sensor-based technologies like an electric field-induced release and measurement (EFIRM) developed by the Wong lab have been shown to detect EGFR mutations (tyrosine kinase domain) from bodily fluids like saliva in patients with non-small cell lung carcinoma (NSCLC) [179]. Similar EFIRM based technologies have been used in developing salivary biomarkers (like *Foxp1 and Gng2*) for the detection of pancreatic cancer [180]. Non-genome-based markers have also been shown to find application in liquid biopsies using saliva. Spectroscopic analysis of salivary metabolites have revealed increased levels of porphyrin to be indicative of oral squamous cell carcinoma (OSCC) [181]. Similarly, changes in the oral microbiome have also been linked by recent studies with the occurrence of OSCC [182].

The completely non-invasive nature of urine sampling, on the other hand, relative to tissue or even blood, makes it a quite useful candidate in LBs, particularly in cases where repeated sampling is required to monitor tumor progression and therapeutic outcomes [183]. Moreover, increasing studies have been directed towards the use of urine in LB due to the presence of a large number of clinically relevant cell-free molecular constituents like proteins, circulating DNA/RNA, and EVs that can be harnessed to monitor tumor oncology. Studies have described urine LBs to be quite useful in detecting cancers of both urological [184] as well as non-urological sources [185] with more or less comparable sensitivities relative to blood LBs [186]. Since most of the biomolecules secreted by cancers of urological origin will most probably be excreted directly into the urinary tract, urine LBs therefore, offer easy and continuous monitoring of these tumors [187]. Some of the already established urinary biomarkers for prostate and bladder cancer include Nuclear Matrix Protein 22 (NMP22), TMPRSS2:ERG fusion gene expression, and Prostate Cancer gene 3 (PCA3) [188] [189]. Urinary lncRNAs, like FR0348383, UCA1, and MALAT1, have been described as biomarkers in PCa even better than similar prostate-specific antigens derived from serum [190–192]. Cell-free nucleic acids like the IQGAP3 and UBE2C present in the urine have been reported recently to act as diagnostic markers for bladder cancer [193]. Non-coding RNAs like piRNA (piR-823), involved in the silencing of transposons, are known to be altered in both serum and urine samples and have been suggested to show promising diagnostic utility in patients with RCC [194]. Moreover, circular RNAs like PRMT5, known to induce epithelial-mesenchymal transition by sponging miRNAs, have been suggested to play crucial role in promoting urothelial carcinoma of the bladder and, thus, positively correlated with advanced clinical stage and reduced survival in patients with urothelial carcinoma [195]. Ongoing clinical trials like NCT04432909 (ClinicalTrials.gov) are underway to further examine urine as a source of LB for urothelial carcinoma. Non-urological cancers like those of lungs, gastric system, colorectal, breast, etc. have also been shown to be diagnosed and monitored with the help of urine LBs. Urinary LBs (like plasma LBs) have been quite successful in detecting epigenetic changes like DNA methylation at discrete gene loci associated with NSCLC [196]. Moreover, sensitivities of tissue and urine LBs were found to be comparable (~ 75%) in detecting EGFR mutations in NSCLC. These and subsequent studies also reported urine LBs to be modest indicators of chemotherapeutic response of tumors against given drugs like Rociletinib and Osimertinib [197, 198]. Recent studies on detection of HCC-associated DNA markers (*TP53 249 T*; *RASSF1A* and *GSTP1* methylations) using urinary LBs have also assisted in monitoring tumor recurrence [199].

## Emerging analytes for liquid biopsies

Tumors have been described as altering platelet behavior in a process termed as tumor-mediated platelet education [13, 200]. Novel biomarkers associated with such TEPs have more recently emerged as promising analytes assisting in the non-invasive detection of cancers [201, 202]. RNA-Seq analyses of platelet-derived RNA were reported to detect both early- and late-stage NSCLC with an accuracy of nearly 80 percent [201]. Furthermore, as tumor development is associated with various systemic changes that lead to metabolic alterations, circulating metabolite levels in the plasma of patients can be exploited as cancer biomarkers. Elevated levels of metabolites, like branched-chain amino acids, have been correlated with the early development of human pancreatic adenocarcinoma [9, 203]. Studies have attributed this to reduced utilization of these circulating metabolites in patients with PCs [203]. In contrast, cancers such as NSCLC that actively utilize these metabolites are associated with reduced levels of branched-chain amino acids. Altered metabolite levels attributed to their differential utilization by tumors might thus play a crucial role in the early detection of various forms of tissue-specific cancers [204].

Moreover, like methylation patterns (discussed before), nucleosome positioning of cfDNA has also been recently described to differ between various cells and offer valuable information regarding target genes. Studies have developed genome-wide maps of nucleosome occupancy on cfDNA by deep sequencing cfDNA samples from blood plasma [205]. Nucleosome occupancy/ ‘footprints’ of cfDNAs may be correlated with the expression of various target genes such as cancer drivers and providing critical information of its tissue of origin [205, 206]. More recently, studies have shown a positive correlation between a decrease in nuclear cfDNA levels and a transition to longer fragments, and an improved chemotherapeutic response in patients with CRC [207]. Conversely, increased and shorter nuclear cfDNA content correlated with tumor recurrence [207]. cfDNA analysis, thus, offers promising breakthroughs in non-invasive liquid biopsy technologies [100]. Circulating cell-free miRNAs associated with EVs, apoptotic bodies, lipoproteins or AGOs (miRISC constituents) are known to be quite stable in a wide variety of body fluids like plasma or serum [208]. Current studies have increasingly focused on using miRNA signatures of body fluids as diagnostic tools in cancer detection. ‘miR-Test’ developed by Montani et al. analyzes serum miRNA signatures and is quite successful in the screening of early LCs, albeit in high-risk individuals relative to traditional and expensive methods such as computed tomography [209]. Similar clinical trials have suggested the significance of plasma miRNA signatures in improving the diagnostic and prognostic evaluation efficacy of LC patients [210]. Therefore, the evaluation of miRNA signatures in body fluids is an emerging area of liquid biopsies that might have huge applications in diagnosing a wide variety of cancers. The evolving list of analytes that can be exploited in liquid biopsies provide novel biomarkers that can find immense application in diagnosis, prognosis, and devising therapeutic regimens for a wide variety of cancers.

## Limitations of liquid biopsies

Recent developments in non-invasive diagnostic and monitoring tools such as LBs have gained widespread attention in cancer treatment. However, LBs are not yet considered a standard tool for confirming and diagnosing different diseases, including cancer, and are primarily used as a complementary test to tissue biopsy. The major limitation of LB is the lack of sensitivity and precision to identify various tumor types compared to tissue biopsy. Moreover, it is also unclear whether LB provides a representative sampling of all genomic clones within an individual tumor or a specific sub-region of the tumor. It is pertinent to mention that diverse features of clonal evolution of tumors such as development of drug resistance, changes in genome, gene expression, epigenetics have been to a great extent successfully addressed by methods such as Single-Cell analysis of cancer cells [211–213]. In addition, the number of CTCs, ctDNA, RNA, progenitor and mature endothelial cells, and tumor-educated platelets are relatively rare compared to other hematological components in the blood, which makes the detection ability of LBs challenging [214].

Moreover, a cost-effective pre-profiling strategy is required for pre-selecting the patients due to the low frequency of target mutations (in the case of CTCs and ctDNA) found in a cohort of patients[215]. Furthermore, some of the biomarkers identified through LB are “fragile” and are difficult to capture. In addition, highly specific and sensitive methods are required to isolate plasma, and there is a lack of standardized methods or protocols for isolation and interpretation [216]. Another limitation observed with LBs is the occurrence of false-positive and false-negative results, which can interfere in correctly evaluating the efficacy of pharmacological treatment [217]. Moreover, the release of biological materials (such as urine and blood) used for LBs can be influenced by microenvironmental factors [217].

The three typical detection targets of LB are CTCs, ctDNA, and EVs, of which EVs are challenging to go from bench to bedside because of the lack of effective enrichment technologies and precise analysis methods [218]. Higher morphological heterogeneity and count of CTCs in different cancers and patients poses a major challenge to LBs. Usually, enriched CTCs are identified using specific tumor-associated biomarkers at either the protein or mRNA level. In patients with epithelial tumors, the identification of epithelial markers can be difficult due to their downregulation during the process of epithelial to mesenchymal transition (EMT), which can lead to false-negative results [219]. In addition, usually, a high starting concentration of CTCs is required for the downstream analysis of individual or clusters of CTCs at the DNA, RNA, or protein level, using micromanipulation or DEP-array technologies [220]. Although whole-genome amplification (WGA) can be employed to generate sufficient quantities of DNA required for sequencing analysis, this technique can induce bias by distorting the initial template during the amplification process, and therefore new WGA-free techniques are required to increase the reliability of these assays [221]. The establishment of permanent CTC cell lines or the development of xenograft models can allow a deeper insight into the CTC-blood microenvironment interactions, but the low number of CTCs in the blood and the heterogeneity of tumors make the establishment of cell lines and xenograft models extremely challenging [222, 223]. Moreover, the development of these models requires a great deal of time, has lower efficacy rates and is not cost-effective, making them unsuitable for clinical investigation.

Another limitation is the low specificity of ctDNAs due to the presence of cfDNAs from normal tissues, which can lead to false-positive or false-negative results [218]. Moreover, to avoid the increase of non-tumoral cfDNA, additional pre-analytical steps are required for the analysis as the cfDNA released from normal cells acts as a diluent for the small fraction of ctDNA and can lead to an inaccurate sampling of cfDNA [224]. Optimized pre-analytical steps such as double plasma centrifugation, proper incubation time, ambient temperatures, and special blood collection tubes for cfDNA can reduce the background of wild-type DNA and provide reliable testing results [225, 226].

Moreover, the establishment of non-coding RNA signatures in the blood is more challenging than other common biomarkers due to their low abundance in body fluids, lack of suitable housekeeping non-coding RNA reference analytes, and high intra-patient variability. These limitations lead to a lack of consistency between biomarkers identified in different studies [227]. Laboratories performing LB assays might also need an initial histological examination by tissue biopsy to avoid over-interpretation of the diagnostic data.

Undoubtedly, LB is an efficient non-invasive diagnostic method that can provide comprehensive tumor-molecular profiling and real-time information on therapeutic cancer targets, but there is a need to develop novel techniques and standardized approaches to overcome the limitations that hamper the implementation of LB into translational and clinical practice.

## Conclusion and future directions

The present literature supports the validity of LB as a minimally invasive diagnostic tool for the early diagnosis and monitoring of therapeutic response, cancer screening in high-risk populations, assessment of tumor heterogeneity, and detection of novel cancer driver mutations. Like tissue biopsy, LB provides molecular tumor information that can allow early detection of tumor burden long before the conventional tests can. Also, LB has great potential to monitor the intratumor heterogeneity, determine the clonal nature of driver events and evolutionary processes in different early-stage cancers, and also serve as a predictive marker for occult metastasis. Moreover, the molecular characterization of tumors unraveled using LB can track disease evolution and prevent disease relapses. Despite the myriad benefits, the clinical application of LB is hampered due to some of its limitations, such as lack of specificity and sensitivity, lack of diverse standardization and isolation procedures, and elevated economic costs. Current technology only provides knowledge of tumor activity and gene expression at a superficial level. There is a need to improve technology that allows multi-organ cancer detection and in-depth tumor analysis. Thus, addressing the challenges associated with the use of LB through advances in research and technology can allow its optimal integration in clinical settings, leading to a profound change in cancer research.

## Data Availability

Not applicable.

## Abbreviations

LB: Liquid biopsy
CTCs: Circulating tumor cells
ctDNA: Circulating tumor DNA
cfRNA: Cell-free RNA
OS: Overall survival
OSCC: Oral squamous cell carcinoma
BC: Breast cancer
CL: Colon cancer
CRC: Colorectal cancer
PCa: Prostate cancer
PC: Pancreatic cancer
ESCC: Esophageal squamous cell carcinoma
RCC: Renal cell carcinoma
OVAC: Ovarian cancer
HEPC: Hepatocellular carcinoma
LC: Lung cancer
NSCLC: Non-small-cell lung cancer
*AR-V7*: Androgen receptor splice variant-7
*Bcl-2*: B-cell lymphoma 2
WBCs: White blood cells
RBCs: Red blood cells
NGS: Next-generation sequencing
WGA: Whole genome amplification
*EGFR*: Epidermal growth factor
TAm-Seq: Tagged-amplicon deep sequencing
CNAs: Copy number alterations
MSP: Methylation-specific PCR
CSF: Cerebrospinal fluid
TEPs: Tumor educated platelets
PARE: Personalized analysis of rearranged ends
TAm-Seq: Tagged amplicon deep sequencing
CAPP-Seq: Cancer personalized profiling by deep sequencing
Safe-SeqS: Safe sequencing system
